# Economic Analysis of a Pediatric Ventilator-Associated Pneumonia Prevention Initiative in Nicaragua

**DOI:** 10.1155/2012/359430

**Published:** 2012-02-08

**Authors:** Edward I. Broughton, Sergio R. López, María Nela Aguilar, María Mercedes Somarriba, Magaly Pérez, Nieves Sánchez

**Affiliations:** ^1^University Research Co., LLC and EnCompass LLC, 7200 Wisconsin Avenue No. 600, Bethesda, MD 20814, USA; ^2^Componente Materno Infantil, University Research Co., LLC, De La Vicky 1, Cuarda al Oeste 1, Edificio Plaza San Ramon, 2° Nivel, Puerta 7, Managua, Nicaragua; ^3^Comité de Infecciones Intrahospitalarias, Infantil Manuel Jesus Rivera Hospital, Barrio Ariel Darce, Managua, Nicaragua; ^4^Departamento de Neonatología, Bertha Calderón Hospital, Del Centro Comercial Zumen 1, Cuadra al Oeste, Managua, Nicaragua

## Abstract

We performed an economic analysis of an intervention to decrease ventilator-associated pneumonia (VAP) prevalence in pediatric intensive care units (PICUs) at two Nicaraguan hospitals to determine the cost of the intervention and how effective it needs to be in order to be cost-neutral. A matched cohort study determined differences in costs and outcomes among ventilated patients. VAP cases were matched by sex and age for children older than 28 days and by weight for infants under 28 days old to controls without VAP. Intervention costs were determined from accounting and PICU staff records. The intervention cost was approximately $7,000 for one year. If VAP prevalence decreased by 0.5%, hospitals would save $7,000 and the strategy would be cost-neutral. The finding that the intervention required only modest effectiveness to be cost-neutral and has potential to generate substantial cost savings argues for implementation of VAP prevention strategies in low-income countries like Nicaragua on a broader scale.

## 1. Introduction

Ventilator-associated pneumonia (VAP), defined as pneumonia occurring more than 48 h after the initiation of endotracheal intubation and mechanical ventilation, is the cause of significant morbidity and mortality in critically ill patients [1, 2]. While the incidence of VAP among adults is higher than among children, pediatric VAP is associated with a 4-fold increase in intensive care unit (ICU) length of stay, a 3-fold increase in hospital length of stay, and higher mortality [3]. 

Implementation of evidence-based quality improvement (QI) strategies to reduce the incidence of VAP in intensive care patient populations has become a major focus in high-income countries, and several authors have outlined clinical management bundles specifically for preventing VAP in pediatric patients [4, 5]. One study conducted in the USA reported the business case for decreasing VAP in PICU settings [6]. However, there has been little research into such efforts in low-income country settings [7].

The incidence of VAP among pediatric patients has been reported at between 16 and 53 cases per 1,000 ventilator-days in Nicaraguan hospitals [8]. This is higher than the 10 per 1,000 ventilator days in Taiwan and the 3.7 per 1,000 ventilator days in the USA [9, 10]. A QI intervention was implemented in two hospitals in Managua, Nicaragua, by USAID's Health Care Improvement Project (HCI) in partnership with the Ministry of Health (MINSA). The goal of the intervention was to decrease the risk of pneumonia among pediatric patients on mechanical ventilation. This study is an economic analysis of the QI intervention to decrease VAP prevalence in PICUs in the two hospitals from the perspective of MINSA and HCI. Specifically, we address three questions: (1) how much does the QI intervention cost to implement?, (2) how effective does the intervention need to be in order to be cost-neutral?, and (3) what are the most important variables in the model?

## 2. Methods

### 2.1. Design and Sampling

We conducted a small matched cohort study to determine the difference between ventilated pediatric patients who developed VAP and those who did not in terms of lengths of stay, case fatality ratios, antibiotic usage, and diagnostic tests conducted. Fifty patient charts were reviewed at the two participating hospitals: 25 cases and 25 controls were matched on sex and age within 30 days for children older than 28 days and on weight within 1,000 grams for neonates under 28 days of age. Fifteen consecutive cases and 15 controls were sampled from Bertha Calderon Hospital, while ten consecutive cases and ten controls were sampled from Manuel de Jesus Rivera Children's Hospital.

### 2.2. Intervention

An initial evaluation identified care practices that did not meet evidence-based standards of care. The intervention involved an improvement specialist working with front-line PICU staffs working as “quality improvement teams” to identify specific deficiencies in clinical practice, implement changes, and evaluate their effectiveness in achieving compliance with care standards. Specific interventions implemented by the teams included the correct use of antiseptics and disinfectants, proper hand hygiene before handling the patient or ventilator equipment, frequent oral care for the patient, draining the ventilator circuit of condensate frequently, and positioning the patient with the head of the bed elevated at least 30 degrees. Team members were also responsible for monitoring compliance with MINSA infection prevention standards [11] and indicators of VAP as well as other nosocomial infections in their facilities. A coach from HCI met monthly with the QI teams to analyze results of the training on hygiene and infection prevention behavior change and propose actions when suboptimal performance was identified. A standardized treatment algorithm to reduce aspiration of secretions was created and adopted by the MINSA as the standard for use in ICUs. QI teams from the two hospitals met periodically to share the results of the changes in procedures of care they had implemented.

Planning and development of the program and coaching personnel were funded by HCI. MINSA covered the costs of the clinical staff's time and any additional expenses involved with implementation of the intervention, such as additional antiseptic supplies. The Nicaraguan MINSA gave approval for this intervention and its evaluation.

### 2.3. Data Collection

Data on lengths of hospitalization, discharge status, use of antibiotics, and diagnostic tests were obtained from a retrospective review of the hospital charts for the cases and controls sampled from the two hospitals.

Data on the costs of the QI intervention were collected from a review of HCI records and approximations made by HCI personnel of the time spent by the participating hospital staff at the coaching sessions. The costs paid by HCI for implementation of the intervention were determined by examining the project's accounting records and include the HCI staff time required to design and implement the QI intervention, travel expenses, administration, and other expenses. Costs to MINSA for the intervention were calculated based on the time they devoted to the activities of the intervention described above, even though these tasks were completed within their normal work day and did not incur additional expenditure.

Hospitalization costs were obtained by summing salaries of doctors and nurses required to staff the ICU, multiplying it by the proportion of patients under care for VAP, and calculated according to the salary rates published by MINSA. Hospital bed costs, which included food, water, medical supplies, electricity and other utilities, cleaners, and ancillary staff, were calculated based on the rates quoted by administrators from both hospitals and divided by the number of patients in care to obtain a per-patient cost figure. The cost of diagnostic services and antibiotics prescribed and consumed during hospitalization was calculated based on the MINSA price list of basic medicines, using the defined daily dose methodology described by Maxwell et al. [12]. We calculated the costs of hospitalization based on the methodology developed by Pan American Health Organization [13]. All costs were calculated based on information provided by administrators of the two hospitals and reported in 2008 US dollars.

### 2.4. Analysis

**Figure 1:**
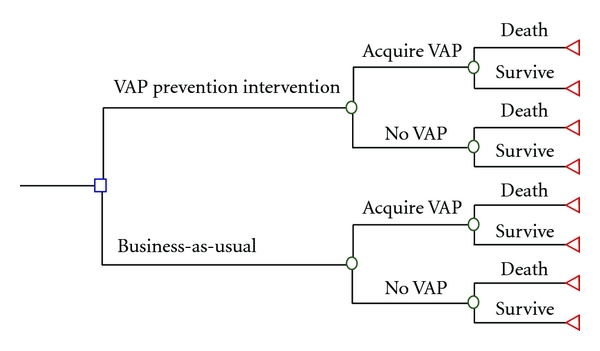
Analysis framework for comparing the QI intervention to business-as-usual.

We used the decision tree analysis to compare the costs and effectiveness of the two strategies: with the QI intervention and business-as-usual (Figure 1). Three outcome measures were used: disability-adjusted life years (DALYs) averted, hospital days averted, and deaths averted. For DALY calculations, a 3% per annum discount rate was applied, and age and disability weighting was used according to the methods described by Murray and Lopez [14] and used for pediatric ICU cases in developing countries by Profit et al. [15]. We used a one-month length of illness for children surviving VAP and a three-week length of illness for non-VAP ventilated children. This was based on the averages determined from the matched cohort study. 

The outcome was measured as an incremental cost effectiveness ratio (ICER), the formula for which is given in (1). A negative numerator means that the intervention costs less. A negative denominator means that the intervention was more effective. If both the numerator and denominator are positive, the intervention saves money and improves health and is therefore strongly recommended.

We were not able to determine the effectiveness of the QI intervention in decreasing the incidence of VAP due to the absence of baseline data. Consequently, a range of levels of effectiveness were entered into the decision tree model to determine how the incremental cost effectiveness changed. From these calculations, the point at which the cost savings due to fewer cases of VAP among ventilated ICU patients were equal to the cost of the QI intervention was determined. Effectiveness at a level higher than this cost-neutral point would lead to both lower costs and better health outcomes, defined as a decrease in the incidence of VAP in this population.

(1) $$\begin{array}{cc} & \text{Incremental}\;\text{cost}\;\text{effectiveness}\;\text{ratio}\\ & \;=\frac{\text{cost}\;\text{at}\;\text{QI}\;\text{sites}\;\text{after}\;\text{intervention}-\text{cost}\;\text{at}\;\text{QI}\;\text{sites}\;\text{before}\;\text{intervention}}{\text{Length}\;\text{of}\;\text{stay}\;\text{at}\;\text{QI}\;\text{sites}\;\text{after}\;\text{intervention}-\text{length}\;\text{of}\;\text{stay}\;\text{at}\;\text{QI}\;\text{sites}\;\text{before}\;\text{intervention}}. \end{array}$$

To determine the relative importance of the input variables, we increased their values individually in turn by 1%, recalculated the model and recorded the effect this had on the outcome. The higher the percent change in the outcome, the more important the change in the input variable.

## 3. Results

The costs of the intervention are given in Table 1. ICU staff time for the intervention was based on a two-person team (one doctor and one nurse) in each hospital devoting 3 hours to QI intervention activities every month. Although this did not create additional expense for the hospitals, their time on QI activities is valued because they were unable to fulfill their clinical duties during this period. The total cost for the year-long intervention was $6,682. The cost of the coaching visits to the two hospitals conducted by HCI Project personnel was the highest expense, accounting for 73% of the total.

Data from the matched control study are given in Table 2. The average number of days of hospitalization was 17 days higher among patients with VAP than those with no VAP diagnosis (*P* < 0.001). There was a 40% higher case fatality ratio among patients with VAP (*P* = 0.02). The average number of laboratory cultures was also higher by 2.9 or 96% among VAP patients (*P* = 0.01). The average cost of hospitalization for a patient with VAP was $9,686, while the average cost for a non-VAP ventilated patient was $3,779.

**Figure 2:**
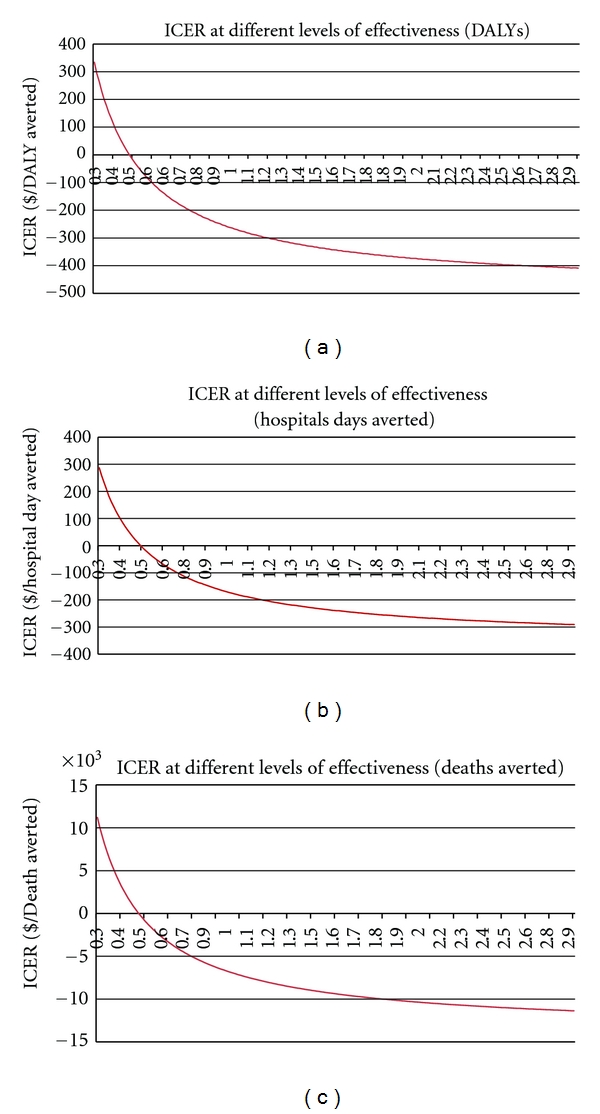
ICER of the QI intervention at different levels of effectiveness.

We modeled the ICERs of the QI intervention for various levels of effectiveness from a decrease of 0.3% in the prevalence of VAP to a decrease of about 3% for the denominators of DALYs, hospital days averted, and deaths averted (Figure 2). For all outcome denominators, the break-even point occurs when the effect of the QI intervention is a decrease in the prevalence of VAP by 0.5%, that is, if the effect of the QI intervention was to decrease the prevalence from 227 cases per 1000 ventilated patients to 222 cases per 1000. There would be increasingly large savings to the health system as the effectiveness of the intervention gets progressively higher than the 0.5% reduction in VAP prevalence. As shown in the three graphs in Figure 2, the ICERs appears to reach a point of diminishing returns in terms of cost savings due to the QI intervention at an effectiveness level of around a 2.5% decrease in the prevalence of VAP. 

The length of hospitalization for VAP cases had the greatest effect of all of the input variables on the relative cost effectiveness of the intervention, with a 1% increase in length of stay causing a 1.82% increase in the ICER. Variables that had little effect on the cost effectiveness result include the cost of antibiotics, the number and cost of laboratory cultures, and the cost of the QI intervention (Table 3).

## 4. Discussion

For an investment of less than $7,000 for the development and implementation over 1 year, the QI intervention would be cost-neutral if it decreased the prevalence of VAP by 0.5%, that is, resulted in 5 fewer cases of VAP per 1,000 mechanically ventilated patients in ICU. If the intervention decreased the prevalence more than 0.5%, there would be significant cost savings as well as improved health outcomes.

The prevalence of VAP in our study population was 22.7%, which is comparable to the prevalence of 20% found in the Punjab [17]. The average length of stay for a VAP patient of 27.9 was similar to the 22 to 37 (depending on the management protocol) found in Taiwan [9]. Taira et al. [18] found lengths of stay for VAP and non-VAP ICU patients of 28.7 and 12, respectively, which was very close to the lengths of stay seen in this study. The magnitude of the cost difference for hospital care between VAP and non-VAP of $5,907 was slightly higher than difference of $4,890 found in Argentina from data collected in 2001 [19]. It was less than the $30,000 difference found in a Unites States (USA) hospital. However, considering the substantially lower expenditures on health and the lower cost of labor in Nicaragua compared with the USA, our results represent a greater practical difference [20].

The average length of stay for VAP cases was the variable in the model that had the greatest impact on the overall result. The average length of stay for non-VAP cases and the case fatality ratios of both VAP and non-VAP cases also had a significant impact on the result. This is consistent with the finding of a large difference in case fatalities and lengths of stay between the cases and controls found in the study. The finding that the cost of the QI intervention itself had only a small impact on the overall cost effectiveness suggests that even if the QI intervention costs substantially more to implement, its efficiency would decrease by only a small degree.

The finding of only a small improvement needed to achieve cost savings is consistent with the findings of Sharma et al. [17] who reported that increased compliance with evidence-based prophylactic measures led to a decrease in VAP cases with an associated significant cost savings among adult ICU patients.

Prior to the QI intervention, the participating ICUs did not record the occurrence of VAP among all mechanically ventilated patients in the unit. The incidence of VAP was not distinguished from infections from other causes, and it was impossible to distinguish VAP cases from other ICU-acquired infections ex post facto in this case due to limitations in patient clinical records. Therefore, baseline data, from which the effectiveness of the intervention could be measured by comparing to data collected at the end of the QI intervention, were not available. Because changes in infection control practice were made rapidly at the onset of the intervention, it was considered invalid to use the data of occurrence on VAP collected concurrently at the beginning of the intervention. If MINSA decides to expand the QI intervention to other ICU facilities in Nicaragua, we recommend that more attention be paid to collection of valid baseline data so that changes in the occurrence of VAP associated with implementation of the QI intervention can be quantified.

Other researchers have quantified the changes in occurrence of nosocomial infections associated with improved compliance with hand hygiene standards. Taira et al. [18] found a decrease in nosocomial infections in a hospital-wide intervention in Switzerland from 16.9 to 9.9% over the course of a year. Acosta-Gnass et al. [19] found a reduction in the incidence of VAP from 28 cases per 1,000 ventilator days to 13.2 cases in the USA If the impact in reduction of VAP incidence in the participating hospitals in Nicaragua was anything close to the impact shown in these other studies, the cost effectiveness of the QI intervention would be substantial. Given that the reduction in prevalence of VAP needed for the cost savings to be equal to the cost of the QI intervention is only 0.5%, we consider it highly probable that this intervention was associated with significant positive outcomes for the participating hospitals.

## 5. Limitations

This analysis took the narrow perspective of the funders (USAID and MINSA) and did not include costs to the patient's family or caregivers as would have been the case if the societal perspective had been taken. The costs to caregivers would be lower if the incidence of VAP decreased as expected, making the QI intervention even more cost-effective than these results.

We were unable to control for confounding factors such as the patients' preexisting health status in the matched cohort study. It is plausible that patients who develop cases of VAP are in poorer health before infection than those who do not develop VAP, and may therefore have worse outcomes regardless of VAP infection. Accounting for this factor would possibly decrease the measure of cost effectiveness seen here. A study with a much larger sample size is required to control for such confounders. Matching patients by pediatric risk of mortality (PRISM) scores or pediatric index of mortality (PIM) scores would also have helped account for differences in preexisting severity of illness. However, the use of these scores is not routine in Nicaraguan PICUs and their validity and reliability have been challenged in some studies [20, 21]. The differences between VAP and non-VAP mechanically ventilated PICU patients in this study were consistent with those of a larger study in which severity score differences were accounted for [3].

This study was conducted in the two largest pediatric hospitals in Nicaragua. They have greater resources, higher patient numbers, and larger staff than other hospitals in the country. It is possible that implementation of the intervention at these other hospitals may involve different per-patient costs and result in different levels of effectiveness. Ideally, sampling across other hospitals in Nicaragua would have supported the case for generalizability of the findings. However, given the simple nature of the intervention, we assume that it could be implemented with similar costs and effectiveness.

The QI interventions studied here was a time-bound strategy designed to implement changes to bring about improvements in the processes of care to ultimately achieve better health outcomes and then to make these improvements sustainable beyond the life of the intervention. If improvements are institutionalized and the QI teams disband or move on to address other gaps in quality performance, the intervention's cost effectiveness will increase in subsequent periods compared to the result shown here.

This is the first economic evaluation of which we are aware that examines the costs and efficiency of a QI intervention for nosocomial infection prevention in tertiary care in a low-income country. The incidence of VAP in developing country ICUs is reported to be four times higher than ICUs in the USA [10]. The need for effective interventions to decrease the occurrence of VAP in ICUs is clear, and determining their cost and efficiency is crucial for informing health policy decision-makers on the affordability and sustainability of the strategies in comparison to competing needs for health resources. This study indicates that modest inputs for the QI intervention are likely to produce significant cost savings even with only a modest decrease in the prevalence of VAP. We, therefore, recommend implementation of this QI intervention in all pediatric ICUs in Nicaragua. However, more data on effectiveness of the intervention in decreasing the occurrence of VAP should be added to the analysis when it becomes available.

## Figures and Tables

**Table 1:** Costs of the QI intervention and ICU inputs.

Cost items		Cost ($US)
QI intervention (for 1 year)	Development	922
	Coaching	4860
	Management	540
	Hospital staff time	360

	Total	6682

Antibiotics per day (VAP)		4.41/day
Antibiotics per day (non-VAP)		1.69/day
Cultures		4.88
Hospital bed (ventilated patient)/day		343

**Table 2:** Noncost inputs for cost effectiveness analysis model.

Inputs	Estimate	Source
Prevalence of VAP among ventilated patients	0.227	Matched cohort study
Case fatality ratio (CFR) for VAP cases	0.5	Matched cohort study
CFR for controls	0.1	Matched cohort study
Average length of stay (LOS): VAP case in days	27.9	Matched cohort study
Average LOS: controls in days	10.9	Matched cohort study
Average number of cultures: VAP cases	5.96	Matched cohort study
Average number of cultures: controls	3.04	Matched cohort study
DALYs severe pneumonia (VAP)	0.056	Hsieh et al. [9]
DALYs severe non-VAP illness	0.037	Hsieh et al. [9]
DALYs death	30.5	Hsieh et al. [9]
Life expectancy Nicaragua in years	73	PAHO [16]

**Table 3:** Effects of a 1% increase in input variables on incremental cost effectiveness ratio.

Variable	% Variation	Direction (cost effectiveness of QI intervention)
LOS (VAP)	1.82	Increase
CFR for VAP	1.33	Increase
Cost of hospital bed	1.13	Increase
Length of stay (non-VAP)	0.60	Decrease
CFR (non-VAP)	0.22	Decrease
Incidence of VAP postcollaborative	0.22	Decrease
Cost of antibiotics	0.10	Increase
Number of cultures (VAP)	0.08	Increase
Cost of cultures	0.08	Increase
Number of cultures (non-VAP)	0.07	Decrease
Cost of QI intervention	0.07	Decrease
